# Selective Attention in Multi-Chip Address-Event Systems

**DOI:** 10.3390/s90705076

**Published:** 2009-06-26

**Authors:** Chiara Bartolozzi, Giacomo Indiveri

**Affiliations:** 1 Robotics, Brain and Cognitive Sciences Department, Italian Institute of Technology (IIT), via Morego 30, I-16163 Genova, Italy; 2 Institute of Neuroinformatics, University of Zurich and ETH Zurich, Winterthurerstrasse 190, CH-8057 Zürich, Switzerland; E-Mail: giacomo@ini.phys.ethz.ch

**Keywords:** analog VLSI, subthreshold, selective attention, multi-chip system, Address-Event Representation (AER), saliency-map, winner-take-all (WTA)

## Abstract

Selective attention is the strategy used by biological systems to cope with the inherent limits in their available computational resources, in order to efficiently process sensory information. The same strategy can be used in artificial systems that have to process vast amounts of sensory data with limited resources. In this paper we present a neuromorphic VLSI device, the “Selective Attention Chip” (SAC), which can be used to implement these models in multi-chip address-event systems. We also describe a real-time sensory-motor system, which integrates the SAC with a dynamic vision sensor and a robotic actuator. We present experimental results from each component in the system, and demonstrate how the complete system implements a real-time stimulus-driven selective attention model.

## 1. Introduction

Selective attention is one of the most powerful strategies used by biological systems, from which robotics, and in general all artificial computation, can take advantage. In a biological sensory system, selective attention acts as a dynamic filter to select the most salient regions of the input and sequentially allocate computational resources so as to analyze them in detail. This serial strategy reduces the computational load and optimizes the usage of the available computational resources.

Here, we present a hardware multi-chip model of visual selective attention, based on a Selective Attention Chip (SAC), which is implemented using neuromorphic analog/digital VLSI circuits. The multi-chip system is composed of a Dynamic Vision Sensor (DVS) [1], the SAC, and a motorized pan-tilt unit capable of orienting the DVS towards salient stimuli. The proposed system can be used as a real-time tool for validating different theories of visual attention, and demonstrates how the SAC can be considered as an attractive building block for implementing attention mechanisms in autonomous robotic platforms and to actively explore the environment in real-time.

The computational primitives implemented in the VLSI multi-chip system use the principles inspired by those of biological neural systems to process visual information. The rationale behind this approach stems from the observation that biological systems and analog VLSI circuits share many similar physical properties, and at the same time face similar constraints: both need to perform with limited resources and cope with intrinsic inhomogeneities of substrate, failures of components or changes in the environment.

Neural systems developed energy-efficient solutions and computational codes [2] that cope with variability and noise by dynamically changing the properties of single computational units [3]. This property makes neural computation robust and fault tolerant, and naturally leads to systems that learn and adapt to their environment [4]. By mapping onto silicon such types of computational principles, neuromorphic engineering aims to produce novel types of compact, low power and smart devices capable of efficiently interacting with the real-world in real-time.

The main contribution of the proposed work is the realization of the first fully functional multi-chip model of visual selective attention, which couples the SAC with an active vision system capable of orienting its gaze towards the most salient object in its field of view. In the following sections we describe the visual attention system’s overall architecture (Section 2), and the properties of its two main components: the Dynamic Vision Sensor (Section 3) and the Selective Attention Chip (Section 4).

An additional important contribution of this work is the improvement of key circuits used in the SAC and the introduction of specific neural computational primitives, such as short time scale types of adaptation mechanisms, that enhance the plausibility and performance of the system. A particular effort was made to thoroughly and quantitatively characterize each circuit in the SAC, when it is embedded in the “behaving” active vision system.

In the following sections we highlight the computational primitives implemented by each of the circuits used in the system: we characterize them at the single chip level behavior (Sections 4.1–4.4), we describe their effects at the system level behavior (Section 5) and we present their response properties in specific application examples (Sections 5.1 and 5.2).

## 2. Visual Attention System

The multi-chip visual attention system implements a model of *bottom-up selective attention* based on the concept of *Saliency Map* [5] - a topographic map where activity encodes for the salience of the corresponding location in the input stimulus, irrespective of the feature that determined the saliency. From the operative point of view, models based on the saliency map allow the read-out of a single unambiguous region of the visual space, corresponding to the maximum of the saliency map, which can be easily used to control actuators. A sequential scan-out of the saliency map, in the order of decreasing salience, determines the shifts of the focus of attention (focus of attention scan-paths). In the case of ocular movements, these shifts determine the end points of saccades, which foveate the selected target and allow for detailed processing of the stimulus.

Computational models of selective attention [6, 7, 8, 9] have focused on the stimulus driven computation that generates the saliency map, and on the mechanisms for creating the attentional scan path from the map itself. A winner-take-all (WTA) competition selects the most salient location in the saliency map, guiding the center of the attentional spot-light. Once selected, a self-inhibitory mechanism deselects the current winner to allow the selection of the second most salient region of the visual field. The iteration of this selection-inhibition cycle induces scanning of the visual field in the order of decreasing saliency, namely the scan-path of selective attention. The number of regions included in the scan-path depends on the duration of the inhibition that prevents re-selection of the most salient stimuli, a phenomenon observed in psycho-physical experiments [10] on visual search and named “inhibition of return” (IOR).

In real world applications such as robot vision, video surveillance, etc., where one of the main requirements is real-time processing and where it is important to reduce the computational load and complexity, software simulations on desktops of such selective attention models are inadequate. Dedicated approaches have been thus proposed with various degrees of flexibility, complexity and computational load, starting from real-time SW implementations run on PC clusters [11] to less sophisticated SW implementation [12] and mixed solutions with software running on dedicated digital hardware (HW) platforms [13].

Another approach is to implement the model on dedicated analog VLSI hardware, that directly maps the theoretical model on silicon. On one hand this approach has less flexibility, since the hardware cannot be fully reprogrammed to implement different models. On the other hand it has the great advantage of real-time and low power operation. Within the neuromorphic engineering approach there are two main streams. One strategy is to implement, on a single chip, the sensors and post-processing that implements a simplified selective attention model using a very small set of features, such as local stimulus intensity [14, 15] or temporal derivative of contrast [16, 17], to compute the saliency map. The second approach, adopted in this work, is to separate the sensory acquisition stage from the processing stage, via the realization of multi-chip hierarchical systems which have the advantage of higher flexibility [18].

The DVS used in our multi-chip system is the one proposed by [1]. This vision sensor responds to temporal variations of light intensity, intrinsically producing a saliency map that encodes for contrast transients. Reference [19] demonstrates that “Motion and temporal change were stronger predictors of human saccades”, as reasonably expected from the behavioural relevance of moving stimuli (prays or predators), with respect to stationary stimuli. In particular, moving and changing stimuli are strong psychophysical attractors of human bottom-up attention, while “static” feature maps (such as intensity, orientation and color) have a very small contribution. Therefore, the DVS will produce a partial saliency map that is nevertheless capable of capturing the most significative information for the orientation of attention.

The sensory data produced by the DVS is transmitted to the SAC for further processing. Specifically, this neuromorphic chip processes the asynchronous events produced by the DVS in real-time, and computes the coordinates of the most salient pixel. It integrates over a tunable time interval the activity of each pixel and compares them, in order to select the pixel that responded to the highest temporal variation of contrast. Each SAC input locally adapts to constant pixel activity from the sensor, filtering out stationary stimuli such as flickering lights often present in our everyday life experience. The SAC implements also a form of self-inhibition that allows to deselect the current winner and move the focus of attention to other stimuli.

The visual attention multi-chip system is realized with the DVS and the SAC by the address event representation (AER) communication scheme [20].

AER is an asynchronous, event-driven, spike-based protocol. Each neuron of a chip transmits the spikes it produces by writing its address on a common digital bus as the spike happens. Information is encoded in the temporal pattern of the events; time is self-encoded and is not explicitly transmitted. When the AER is used to connect two neuromorphic chips with the same communication latency, as in the proposed visual system proposed, the sequence and the relative implicit timing structure of the events are preserved. However, if the communication happens between a neuromorphic chip and a device with intrinsically different information encoding (e.g., a computer), the AER interface can append an explicit time stamp to the address of each event, in order to preserve the real-time information of the emitted spikes.

The proposed visual attention system exploits the AER infrastructure to interface the DVS chip to the SAC chip, and the SAC chip to a robotic pan-tilt unit which is driven by the result of the selective attention competitive processing.

## 3. Dynamic Vision Sensor

The vision sensor used [21] generates asynchronous events that correspond to the temporal changes in the logarithm of local image intensity. As the ratio $\frac{d}{\mathrm{dt}}$ log *I* is equal to $\frac{\mathrm{dI}/\mathrm{dt}}{I}$, where *I* is the pixel illumination, the DVS encodes relative temporal changes in contrast (rather than absolute illumination, encoded by standard image sensors). This property allows the sensor to respond to 20% contrast changes over a dynamic range spanning over 5 decades. In addition, computation is performed independently by each pixel (local gain control), allowing the DVS to optimally respond to scenes with non-homogeneous illumination.

The DVS efficiently processes signals measured from real-world scenes in uncontrolled environments with very wide dynamic range spans, such as outdoors, by exploiting the same elegant solutions used by biological neural systems: local gain control, adaptation and relative rather than absolute computation. These computational strategies are present at all levels of brain computation, from the photoreceptors in the retina to the neurons in cortex [22, 23].

The event-driven nature of the DVS ensures that visual data are only transmitted when pixels sense sufficient contract changes. This reduces redundancy and produces a sparse image coding, therefore optimizes the use of the communication channel and the post-processing elaboration and storage effort. The real-time asynchronous output nature of the DVS ensures precise timing information and low latency [24] yet requires a much lower bandwidth used by frame-based image sensors of equivalent time resolution [25].

Behaviorally, the DVS responds only to relative changes with respect to the static background. In the visual attention system it has the essential role of transmitting information on local change of scene reflectance, producing a saliency map based on moving or changing stimuli. Specifically, each pixel of the retina responds to both positive and negative variations in contrast, transmitted as ON and OFF events respectively. In the implementation of the visual selective attention two-chip system, ON/OFF information is discarded and the events sent to the SAC indicate a variation of contrast, irrespective of its polarity.

## 4. Selective Attention Chip

The post-processing chip computes the scan path of the focus of attention from input saliency maps by using computational primitives analogous to those used by biological neural systems. It is the evolution of previously proposed selective attention chips (see [26] for a review). In addition to incremental improvements to the circuits, the SAC includes short-term depression (STD) circuits [27] and spike frequency adaptation ones [28], which play a crucial role in making the chip sensitive to changes in the input stimuli, and in reducing the amount of information sent to the output bus.

The core of the chip comprises an array of 32 × 32 pixels. Each pixel is 90 × 45 *μ*m^2^. The whole chip with external interfacing circuits and pads occupies an area of 10 mm^2^.

**Figure 1.**
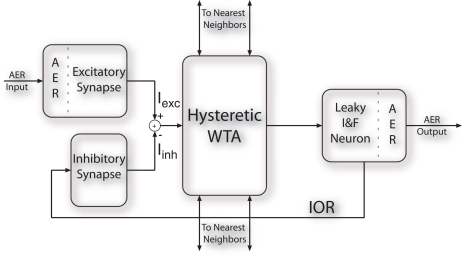
Block diagram of the SAC pixel. Each pixel receives sequences of spikes from the AER bus and competes for saliency by means of lateral connections. The winning pixel sends its address to the AER bus and self-inhibits via the inhibitory synapse.

Figure 1 shows the block diagram of a SAC pixel: each pixel in the array receives as input sequences of spikes encoding for the saliency of the corresponding pixel in the visual stimulus; an input excitatory synapse translates its input spikes into the current *I_exc_*. A current-mode hysteretic WTA competitive cell compares the input currents of each pixel: only the winning cell sources a constant current (independent of the input current) to the corresponding output leaky Integrate and Fire (I&F) neuron [28]. The identity of the spiking neuron signals which pixel is winning the competition for saliency. The output spikes of the I&F neuron are also sent to a feedback inhibitory synapse, that produces a negative current (*I_inh_*) which is subtracted from the input node of the WTA cell. The net input current to the winner pixel decreases, and a new pixel is eventually selected. This self-inhibition mechanism, IOR, allows the network to select sequentially the most salient regions of input images, reproducing the attentional scan path [29].

In the following, we describe each component of the SAC pixel from its circuit “low level” properties to the functional role in the attentive system behavior.

### 4.1. Input Excitatory Synapse

**Figure 2.**
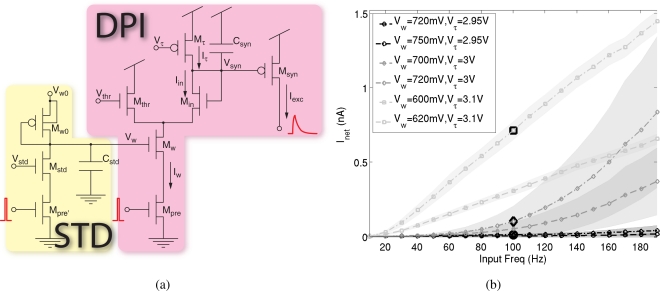
Input excitatory synapse: (a) Circuit diagram of the excitatory synapse, comprising the DPI circuit and the STD circuit. (b) Mean and standard deviation (shaded areas) of the input current of the WTA cell versus the input frequency, when the DPI is stimulated with a spike train of constant frequency at 100 Hz, for different time constant and weight settings and disabled STD.

The silicon synapse used to integrate the input spike trains [30] was designed to support the elaborate dynamic mechanisms observed in selective attention systems. As shown in Figure 2(a), it comprises the differential pair integrator (DPI) that reproduces the currents originated by presynaptic action potentials across the postsynaptic neuron’s membrane, and the STD circuit, that modifies the efficacy of synaptic transmission on the short time scale.

The DPI was explicitly designed to reproduce postsynaptic currents with realistic time course, which can be modeled with exponential functions [31]. Such property is necessary to reproduce another characteristic of synaptic transmission, which is linear summation. In turn, this guarantees that the mean level of the output current of the DPI in response to a train of spikes is linearly proportional to the mean spike frequency, *f̄*:

(1) $$<I_{\mathrm{syn}}>=\frac{I_{w}\;I_{\mathrm{gain}}}{I_{\tau }}\Delta t\bar{f},$$

where *I_w_*, *I_gain_* and *I_τ_* are tunable currents that set the synaptic efficacy and time constant, and Δ*t* is the spike duration.

This is a fundamental requirement for the SAC implementation: the mean frequency of input spike trains encodes for pixel’s saliency, and it is translated into the mean output current produced by the DPI. The WTA competition therefore compares current levels that are directly proportional to the stimulus saliency.

As WTA competition is instantaneous and continuous in time, it is crucial to produce smooth currents that do not have large peaks in correspondence of the input spikes; otherwise the result of the competition would depend on the relative timing of the input spikes, and be independent of their input frequency.

Figure 2(b) shows the mean output current versus the input frequency, when the synapse is stimulated with spike trains of constant frequency. The shaded areas in the figure show the standard deviation of the current (the standard deviation of the mean over time of the current is calculated with the standard procedure for error propagation: if the derived measure is *f*(*x*, *y*, ...), where *x*, *y*, ... are the direct measures, its standard deviation is $\sigma =\frac{\partial f}{\partial x}\sigma_{x}+\frac{\partial f}{\partial y}\sigma_{y}+\ldots$): for long time constants (i.e. *V_τ_* close to the supply voltage) the output current is smoother and the mean current is directly proportional to the input frequency for a wide range of input frequencies.

#### Short Term Depression

STD is a mechanism that modifies synaptic efficacy on a time scale of the order of hundreds of milliseconds to seconds. It is induced by the recent history of the presynaptic firing rate and decreases the efficacy of synaptic transmission for repetitive stimulation. The most evident property of STD is the enhanced response to transients and the adaptation to sustained stimulations, therefore it plays an important role for implementing selectivity to transient stimuli and contrast adaptation [32].

The implementation of this neural computational primitive on the SAC is crucial for enhancing sensitivity to transients: In terms of bottom-up attention, the saliency of stimuli whose attributes never change is decreased, and stimuli with one or more varying attributes become more salient, implementing a form of visual adaptation [33].

### 4.2. Hysteretic Winner-Take-All

The computational core of the SAC is the circuit implementation of a classical functionality observed in neural networks required forand model selective attention: the WTA circuit. In biological neural networks, WTA competition is an emergent computational property of recurrently connected neurons, which enhances the activity of neurons receiving the strongest input and suppresses the activity of neurons receiving weaker input signals. It is one of the competitive-cooperative computational strategies capable of extracting information from noisy and ambiguous data thanks to the evaluation of each stimulus in its own context [34, 35, 36].

**Figure 3.**
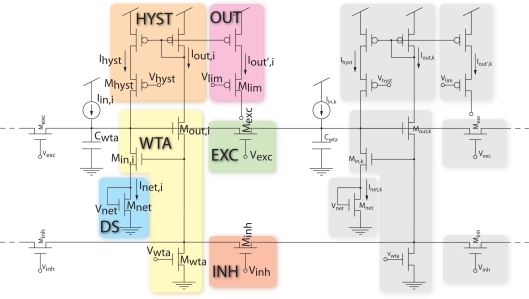
Current-mode hysteretic WTA circuit with diode-source degeneration “DS”, local excitation “EXC”, local inhibition “INH” and positive feedback “HYST”.

Figure 3 shows a bi-dimensional current-mode hysteretic WTA circuit [37] based on a functional model rather than a network of spiking neurons. The proposed circuit is very compact and scalable and operates at low power. Its most striking property is the simple connectivity among units, which allows the realization of dense bi-dimensional arrays with many units on a single chip. In the original current-mode WTA circuit, originally proposed in [38] (“WTA” in Figure 3), the pixels compete for a common resource. The pixel receiving the highest input current can access the resource and output a constant current, while suppressing all other pixels. The additional circuits proposed in this system implement lateral facilitation, local competition and hysteresis. It has been shown that such circuits can significantly improve the performance of the original circuit by speeding up the winner selection, thus increase the network resolution and reduce the effect of input mismatch [37]. In the next paragraphs we show the properties of each circuit as well as the functional role that they have in the complex behaviour of the WTA selection.

#### Hysteresis

The positive feedback “HYST” block of Figure 3 was introduced to further increase the resolution of the circuit and improve its speed [39]. This way, as soon as one cell begins to suppress the others and its output current increases, its input current also increases and the dynamics of the selection speeds up. The feedback current in the input node of the winning cell stabilizes the selection, and implements a form of hysteresis (or short term memory): a new cell can win the competition only if its input current exceeds the input current of the winner plus the positive feedback current *I_hyst_*.

**Figure 4.**
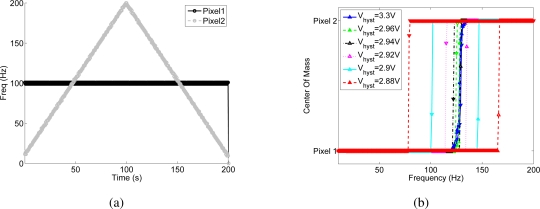
Hysteresis measured by observing the output activity of the I&F neuron: (a) Instantaneous input frequency of the spike train sent to pixel 1, and to pixel 2. (b) Center of mass of the chip’s activity versus the input frequency of pixel 2 for different amplitudes of the hysteretic current.

Figure 4 shows the effect of the positive feedback current on competition between two pixels of the array (namely pixel 1 and pixel 2), quantifying the effect of the bias, *V_hyst_*, that controls the magnitude of the hysteretic current, when the input frequency of pixel 2 increases linearly from 10 Hz to 200 Hz and back with a resolution of 1 Hz and the input to pixel 1 is kept constant at 100 Hz, as shown in Figure 4(a).

Without hysteretic current (*V_hyst_* = *V_dd_*), the WTA should switch from pixel 1 to pixel 2, when pixel 2 receives an input frequency slightly higher than 100 Hz. The WTA should then switch back to pixel 1 when the input frequency of pixel 2 decreases back to a value slightly lower than 100 Hz. When the hysteretic current is enabled, the winning pixel receives an extra current in the input node, this is equivalent to having an input spike train to the winner of frequency at (100 + Δ*f*) Hz, where Δ*f* depends on the bias *V_hyst_*. In such a case, pixel 2 can win the competition only if its input frequency increases above (100 + Δ*f*) Hz. As pixel 2 is selected by the WTA, the hysteretic current is removed from pixel 1 and is injected in the input of pixel 2. For pixel 2 to be deselected, its input frequency has to decrease below (100 − Δ*f*) Hz.

The hysteretic curves of Figure 4(b) are obtained by plotting the center of mass (*CoM*) of the network activity versus the input frequency of pixel 2, where *CoM* is defined as

(2) $$\mathrm{CoM}=\frac{\sum_{i}\;\theta_{i}\;f_{i}}{\sum_{i}\;f_{i}},$$

where *θ_i_* is the address of the ith pixel and *f_i_* is its output frequency. Depending on the resolution of the WTA network, both pixels are selected as winners for a given range of similar input frequencies, and the center of mass is in between the two stimulated pixels, otherwise the CoM corresponds to the pixel selected by the WTA. When the hysteretic current is disabled (*V_hyst_* = *V_dd_*), the *transition frequency* of the WTA, defined as the input frequency at which the WTA changes from one winning pixel to another, depends on the mismatch between the input currents generated by the excitatory synapses of the two pixels. In the shown experiment the transition point is around 125 Hz, and is the same for increasing and decreasing values of the input frequency of pixel 2. The width of the hysteretic curve obtained by enabling the positive feedback increases with increasing hysteretic current amplitude. This experiment confirms also the circuital role of the positive feedback in increasing the WTA resolution: when the hysteretic current is active, the transition between one winner and the other is sharper than in the baseline, as there is typically only one point in the center of mass of the activity that corresponds to the activation of both pixels. Figure 4(b) shows that the resolution of the WTA without positive feedback is about 15 Hz: within this range the WTA cannot resolve between the two inputs, and both pixels are active. The activation of the feedback current increases the resolution of the network up to 1 Hz.

#### Lateral Facilitation

In Figure 3, the transistors *M_exc_* implement a diffusor network applied to the input node of the WTA, diffusing the total input current to the cell [37] and comprising the hysteretic current. This modification gives a competitive advantage to the pixels close to the winner and allows the network to select and track moving stimuli. In addition to being useful for tracking, the diffusion of the input current implements spatial smoothing, giving a competitive advantage to regions of activity (which in vision typically correspond to objects) compared with single pixels, and reducing the effect of mismatch between pixels.

**Figure 5.**
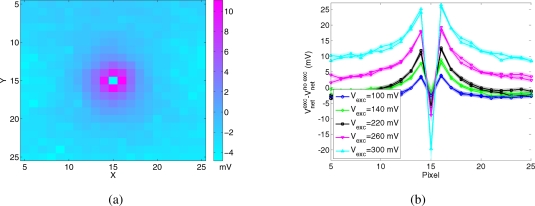
Lateral excitation. Spatial impulse response of the WTA resistive grid (see ”EXC” in Figure 3). The difference between the response for *V_exc_* > 0 and the response for *V_exc_* = *Gnd* is plotted. (a) Example of the spatial response for *V_exc_* = 200 mV, (b) Cross section with mean and standard deviation of the data recorded from the pixels belonging to the same row and column as the central pixel, for different values of the bias *V_exc_*.

To measure the spatial extent of the diffusion of input current for different values of the parameter *V_exc_*, we stimulated the central pixel of the array with a constant spike train at 100 Hz, and measured the voltage *V_net_* of the surrounding pixels. Figure 5 shows the difference between the mean of *V_net_*, obtained for a fixed value of *V_exc_*, and its baseline, obtained when the lateral excitation is disabled (*V_exc_* = *Gnd*). Figure 5(a) shows the data of all recorded pixels, for *V_exc_* = 200 mV. The first neighbors of the stimulated pixel are brighter, then the amount of current received by more distant pixels decreases sharply. Figure 5(b) shows mean and standard deviation of the same data, recorded from the pixels belonging to the same row and column as the stimulated pixel, for a subset of values of *V_exc_*. The value of the central pixel is negative, since the lateral spread of its input current decreases the net current to the pixel itself with respect to the baseline. For high values of lateral excitation the current spreads equally to the limit of the array, and the input loses any information about the location of the stimulated pixel.

**Figure 6.**
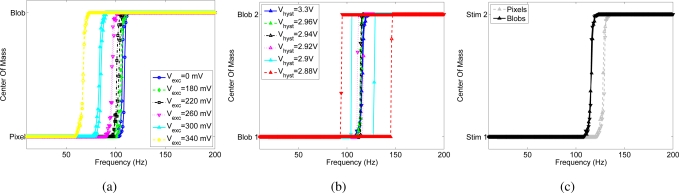
Functional role of lateral excitation. (a) Center of mass of the activity of the array, when stimulating a single pixel with a constant frequency and a blob, for different values of the bias *V_exc_*. The activity of all of the pixels belonging to the blob is added together, and represented as a single pixel. (b) Center of mass of the chip activity, when two blobs are stimulated. (c) Baseline activity without hysteresis, when either single pixels or blobs are stimulated.

The plots in Figure 6 show the functional role of lateral facilitation in the WTA competition. Similar to the experiment used to characterize the hysteresis circuit, pixel 1 is stimulated with a constant frequency at 100 Hz, while pixels belonging to an area of 3 × 3 centered around pixel 2, from now on referred to as *blob*, are stimulated with spike trains with frequencies ranging from 10 Hz to 200 Hz with steps of 1 Hz and back (same frequency profile as shown in Figure 4(a)). Figure 6(a) shows the center of mass of the activity of the chip shifting between the single pixel and the blob for different values of the bias *V_exc_*, and without the hysteretic current. When the lateral excitation is enabled, the pixels belonging to the same blob cooperate and the transition frequency of the WTA selection shifts toward lower values, until the blob wins even when stimulated with a lower frequency than the pixel.

Thanks to the lateral facilitation, a contiguous region of activity (e.g. arising due to an object) has a competitive advantage over a single pixel of activity.

The experiment of Figure 6 unveils an additional role of lateral facilitation, spatial smoothing, that reduces the effect of mismatch between pixels. As pointed out in Figure 4, the transition frequency of the network does not correspond exactly to the input frequency of pixel 1 (100 Hz), but shifts toward a higher frequency because of mismatch between the input currents generated by the two synapses. One way to reduce this effect is to use lateral excitation to smooth the input in space. Figures 6(b) and 6(c) show the same experiment as in Figure 4, stimulating the two *blobs* centered around pixel 1 and 2. The transition frequency of the WTA selection shifts toward the input frequency of pixel 1 (100 Hz), showing a reduction of the effect of mismatch between the input currents to the WTA pixels. The width of the hysteretic curve is smaller, since the hysteretic current spreads also to the neighbors. The slight asymmetry in the hysteretic curves with respect to the baseline transition is probably due to mismatch between the hysteretic currents.

These measures exhibit the functional role of lateral facilitation, showing that a region of activity has a competitive advantage over a single pixel, furthermore, spatial smoothing of the input currents helps in reducing the effect of mismatch in the input synapses.

### 4.3. Output Integrate and Fire Neuron

The output current of each WTA cell is transformed into a train of pulses by a read-out I&F neuron in order to exploit the AER communication system at the output of the chip. It is a convenient and robust method for multiplexing the activity of large networks on a single bus, and necessary for including the SAC in multi-chip systems where we can stack multiple instances of the SAC to build hierarchical models of selective attention. In this scheme the address of the active neuron encodes the result of competition, and in the case of visual selective attention, the position in the visual field at which to deploy attention.

**Figure 7.**
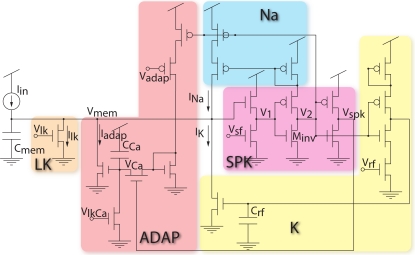
I&F circuit diagram. It comprises a membrane capacitor, a constant leak “LK”, a spike generating circuit “SPK” with a positive feedback “Na” mimicking the fast activation of sodium channels and a reset and hyperpolarizing circuit that mimics the activation of late potassium channels “K”. The “ADAP” circuit implements spike frequency adaptation by calcium accumulation.

The I&F neuron circuit adopted for the SAC is a phenomenological model of a spiking neuron, namely the low-power leaky adaptive I&F neuron model [28], shown in Figure 7. It has tunable leak, refractory period, spiking threshold and implements a mechanism of spike frequency adaptation. The adaptation mechanism reduces the activity of the neurons in periods of prolonged activity. In the SAC application, a constant output firing rate at steady state is not informative, and the adaptation mechanism helps to decrease the number of events produced by each single neuron, which reduces the traffic on the AER bus and thus reduces bandwidth use and power consumption.

In addition to being used for monitoring the activity of the neurons to follow the movement of the focus of attention, the output spikes of each neuron are integrated by the corresponding local inhibitory synapse, which subtracts the current *I_inh_* from the input of the WTA node and implements a self-inhibition mechanism that allows the WTA network to deselect the current winner and select a new one.

### 4.4. Inhibition of Return

The inhibitory synapse provides a mechanism for the deselection of the winning neuron in favor of stimulus exploration. Hardware systems based on the concept of selective attention and WTA competition that lack such deselection mechanism have been specifically designed for tracking a selected target [14, 40]. In such implementations, once a target is selected, the system is designed to lock on the target, disregarding distracters and even new salient stimuli.

The SAC implementation comprises both hysteresis that favors tracking and a self inhibitory mechanism that favors shifts of attention. The dynamic interplay of these two mechanisms creates a complex behavior, mimicking the rich mixture of attentional tracking and shifts of natural scan paths. Similar systems have been proposed in the context of aVLSI implementation of visual selective attention [41, 42].

In the SAC, the inhibitory version of the DPI synapse integrates the spikes of the winning I&F neuron, and generates an inhibitory current *I_inh_* which is subtracted from the input current *I_exc_* until the net input current to the winner is lower than the input currents of the other pixels in the array (see Figure 1). Another pixel can then be selected by the WTA network.

The time course of build-up and decay of inhibition can be tuned by changing the synaptic weights *V_winh_* and *V_thrinh_*, the synaptic time constant *V_τinh_*, and by modulating the firing activity of the I&F neuron.

The scan path of the network depends on the IOR settings, the relative magnitudes of the input currents, and the hysteretic current. The number of selected neurons in the scan path increases with the duration of the inhibition decay. The higher the hysteretic current, the longer the duration of the active period of each neuron.

**Figure 8.**
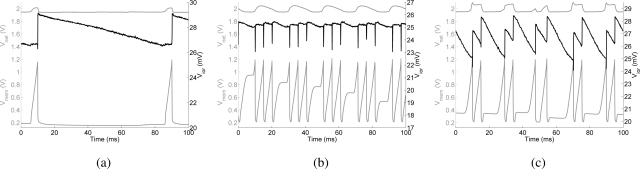
Inhibition of return: typical traces of the internal variables *V_net_* (top trace), *V_mem_* (bottom trace), and *V_ior_* (black middle trace), recorded from one test pixel, for three combinations of inhibition weight (*V_winh_*) and time constant (*V_τinh_*). (a) *V_winh_* = 2.42 V, *V_τinh_* = 10 mV, (b) *V_winh_* = 2.58 V, *V_τinh_* = 30 mV,(c) *V_winh_* = 2.44 V, *V_τinh_* = 80 mV.

The dynamics of IOR can be characterized by measuring the duration of the activation of the I&F neuron and the duration of its suppression. A test pixel (0,31) was stimulated with a spike train of constant frequency of 100 Hz, and the IOR dynamics were evaluated for different values of *V_winh_* and *V_τinh_*. Figure 8 shows the time course of the pixel’s internal variables: The lower trace shows the membrane potential *V_mem_* of the output I&F neuron (see Figure 7). Depending on the bias settings of the inhibitory synapse, one or more spikes are sufficient to inhibit the current winner. The upper trace corresponds to the input node voltage of the WTA *V_net_* (see Figure 3), and shows the decrease of the input current to the current winner due to the inhibitory current. The middle trace shows the gate voltage of the output transistor of the inhibitory synapse. A linear decrease of this voltage results in an exponentially decaying current subtracted from the input node of the corresponding WTA cell. Depending on the time constant and on the weight of the synapse, the output inhibitory current pulse will last from a few milliseconds up to two seconds.

#### Saliency Map

The effect of different IOR settings at functional level on the produced focus of attention (FOA) scan path can be evaluated when stimulating the SAC with saliency maps generated by a software implementation of the algorithm proposed in [7], the Matlab Saliency Toolbox [43], and comparing the properties of the SAC-generated scan path to the scan paths obtained by the software implementation.

The saliency maps were computed from *color*, *intensity* and *orientation* (at 0,45,90,135 degrees) feature maps, weighted equally and summed.

The resulting saliency maps were transformed into an appropriate input for the SAC: for each pixel, a constant spike train was produced whose frequency was proportional to the saliency value of the pixel itself. To have an input range within the linearity region of the input synapses, the saliency values are mapped to an interval between 0 Hz and 200 Hz.

**Figure 9.**
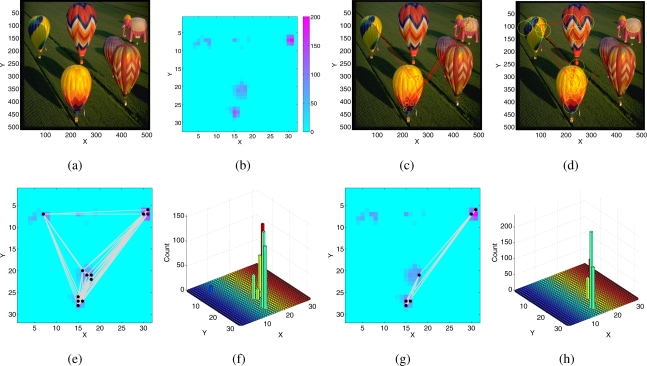
SaliencyToolbox: (a) Input image used for the following experiments (part of the image database of the SaliencyToolbox). (b) Saliency map relative to the input image, obtained from the SaliencyToolbox, with the default parameters. (c–d) FOA scan path generated by the SaliencyToolbox: The yellow circles are centered around each fixation point belonging to the FOA scan path, the red lines connect consecutive fixations. The radius of the yellow circles shows the size of the inhibition area. (c) Small inhibition area (d) Large inhibition area. (e–h) FOA scan path generated by the SAC superimposed on the saliency map for different settings of the IOR. (e–g): black dots show the fixation points, the grey lines connect consecutive fixations; (f–h) histogram of the visited points in the saliency map. (e–f) Slow IOR time constant. (g–h) Fast IOR time constant.

Figure 9(b) shows the corresponding saliency map computed by the SaliencyToolbox with its default parameters. The inhibition region is a disc centered around the FOA. Figure 9 shows the resulting scan path for two different dimensions of the inhibition region. If the size is small (Figure 9(c)), the algorithm chooses many points belonging to the same few objects with high saliency and avoids less salient targets. If the size is bigger (Figure 9(d)) less salient locations are visited.

A similar behavior is also observed in the scan path generated by the SAC when stimulated with the same saliency map. Figure 9(e) and 9(g) show the FOA scan path generated by the SAC superimposed on the saliency map for two different sets of parameters, which differ in the time constant of inhibition. In the SAC, the space constant of the lateral excitation also contributes to the lateral spread of the inhibitory current, but the inhibition region decays exponentially within few pixels and depends on the amplitude of the current itself. Therefore, it is not possible to set arbitrary sizes for the inhibition region. The number of different pixels selected in the scan path, including lower saliency regions, increases if the time constant of IOR is longer: the inhibitory current forces the input to the inhibited pixel to a low value, the network does not select previous winners for a long time, and less salient pixels can be selected. The SAC with global competition, lateral excitation, hysteresis, and IOR, qualitatively reproduces the scan paths generated by the SaliencyToolbox, but selecting regions of higher saliency more often.

The experiments performed here show that we can use the SAC as a tool for implementing the final decision stage of the saliency map model, and use it to explore the parametric space, in real-time. Additionally, it can also be tuned to operate as a local WTA, without IOR, to implement the required local competition and normalization in the feature map component of the saliency map model. Multiple instances of the SAC tuned for different features can be used to build a hierarchical selective attention multi-chip system.

## 5. System’s Behavior

In this section we describe the behavior of the saliency-map based selective attention two-chip system, comprising the DVS as vision sensor, the SAC performing the attentional selection and an actuator that orients the sensor towards the selected salient stimuli. This system is a test bed for evaluating the scan paths generated with real stimuli, and the behavior of the system when it interacts with the real world. It represents a proof of concepts for future miniaturization of the system that can be used in autonomous robots applications for stimulus selection and real-time reaction to external events.

**Figure 10.**
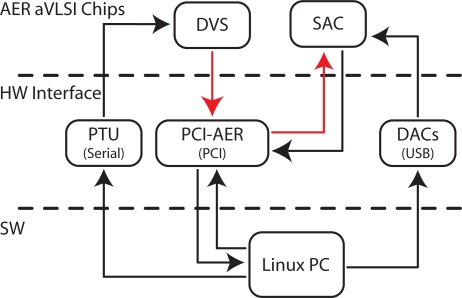
Visual selective attention AER multi-chip system comprising the DVS and the SAC.

Figure 10 shows all of the components of the experimental setup. It comprises the DVS mounted on a Pan-Tilt Unit (PTU), the SAC and a PCI-AER board connected to a Linux PC [44, 45]. The realization of such a complex system was allowed by custom software [46] and hardware infrastructures [45, 47], developed by different groups for AER-based neuromorphic chips. The PCI-AER interfacing board routs the output events from the DVS to the SAC input; thanks to the software interface it can be used to monitor the activity of both chips on a desktop computer for off-line analysis as well as on-line for controlling the PTU. A board comprising an array of digital to analog converters (DAC) is used to set voltage biases and tune the chip’s behavior.

The DVS produces a proto-saliency map based on the information of local luminance changes in time (see Section 3); it has an array of 64 × 64 pixels; its output spikes are routed to the SAC via the PCIAER mapper functionality: a look-up table implements a 4:1 mapping of the DVS addresses onto the 32 × 32 addresses of the SAC. The next physical location of the FOA is determined by a software algorithm, based on the spiking activity of the SAC, acquired via the PCI-AER monitoring functionality. The resulting coordinates of the FOA are transformed into a motor command, and sent to the PTU connected to the PC via a serial port, to shift the central pixels of the retina to the coordinates of the new FOA.

In the first experiment the FOA scan path is recorded while maintaining the retina fixed at its initial fixation point, reproducing what in psychophysics experiments is known as *covert* attention (*Covert attention* is the term used in psychophysics experiments to indicate that the subject performs the task while maintaining his/her eyes fixed, typically looking at a central fixation point. In such experiments attention is measured from increase in performance at the attended location.).

In the last experiment the FOA is used to foveate the retina, reproducing *overt* attentional scan paths [48].

### 5.1. Covert Attention with Short Term Depression - Distinguishing Motion from Repetitive Stimuli

As described in Section 4, the short term form of synaptic adaptation implemented in the input excitatory synapses renders the SAC sensitive to moving and changing stimuli, which are strong psychophysical attractors of attention [19]. This mechanism might appear to be redundant when the input to the SAC is provided from the transient silicon retina chip activity. Nevertheless, in many practical situations there are stimuli that produce activity at a constant frequency, therefore eliciting a strong activity in the DVS (for example flickering lights). However such stimuli are stationary because their frequency does not change in time, therefore they should not be selected as strong attractors of attention.

One approach to overcome this problem is to remove activity corresponding to the stationary stimuli by using a band pass filter [49] tuned to their frequency. Alternatively, the STD mechanism filters out inputs at high constant frequency. Using the STD properties of its input synapses, the SAC can be used to select the activity elicited by transient stimuli, and at the same time suppress the activity of the stationary ones.

**Figure 11.**
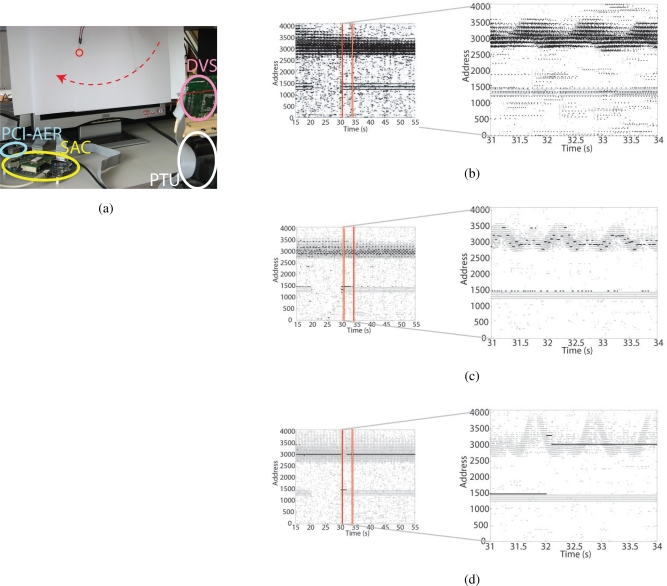
Selective attention multi-chip system: (a) Experimental setup: In front of the DVS, over a white background, a LED (red circle) is flickering and a nut (red dashed arrow) is let oscillating. (b–c) The raster plots show the activity of the SAC (black dots) superimposed to the activity of the DVS (grey dots): Each dot corresponds to a spike emitted by the pixel with address on y-axis at the time indicated on the x-axis. The spikes with addresses ranging from about 1200 to 1500 correspond to the LED evoked activity, the spikes with addresses belonging to the range from ca. 2800 to ca. 3500 correspond to the nut swing. On the left the activity is plotted for 50 s. The right column corresponds to the time zoom over three seconds right after the LED turning on. (b) Baseline experiment: competition, lateral excitation, hysteresis, and IOR, are turned off. (c) WTA competition, IOR (*V_τinh_* = 80 mV, *V_thrinh_* = 200 mV, *V_winh_* = 2.4 V) and STD (*V_wstd_* = 280 mV). (d) WTA competition, IOR turned off, STD (*V_wstd_* = 280 mV).

We reproduced a situation with one stationary stimulus and one transient stimulus, with the setup shown in Figure 11(a): the LED flickering at a constant rate is used as a stationary stimulus, and the oscillating nut represents the “transient” stimulus. The LED is driven by a function generator, at a frequency of 200 Hz. During the experiment the LED is transiently turned off and, after a 10 s pause, turned on again. Figure 11(b) shows the raster plots of the retina (in grey) superimposed on the SAC activity (in black), for the baseline condition (i.e. without competition, lateral excitation, hysteresis, IOR or STD). Each active pixel of the retina elicits spiking activity in the corresponding I&F output neuron. Figure 11(c) shows the SAC response when WTA competition is enabled, as well as the IOR mechanism active. If the STD mechanism is absent (*V_wstd_* = 0 V, not shown), the WTA switches between the two input stimuli, the LED and the nut. When STD is enabled, the LED is selected only for a short transient and the SAC follows the nut movement.

Figure 11(d) shows the SAC response in the same configuration as above, but without IOR: the SAC selects one pixel stimulated by the nut trajectory, the corresponding input frequency is not high enough to elicit a significant depression of the synapse weight, and hysteresis contributes to maintain the selection stable. When the LED is turned on the winner switches transiently, for as long as the weight of its input synapse is not suppressed by the STD. When STD decreases the weight of the pixels stimulated by the LED, the first selected winner is chosen again. Without the IOR mechanism, the SAC does not disengage from the selected pixel, unless a stronger input is applied. This configuration is useful to evidence the transient effect of the appearance of a strong stationary stimulus and highlight the essential role of IOR in dynamic tasks.

The behavior of the system in this experiment shows the high level effect of the implementation of a low-level feature such as STD: stationary inputs do not attract attention, but their transient activation is capable of capturing attention for a short period. Therefore, the system is still sensitive to the appearance of (potentially) behaviorally important stimuli.

The superposition of retina and SAC activity shows a short latency of the SAC response, due to the dynamics of the interaction between IOR and hysteresis. When one pixel is selected, hysteresis keeps it active until the IOR inhibits the pixel and the WTA selects a new winner. In the meantime, the stimulus has moved away, and the SAC follows it with a short latency. The latency could be corrected by modeling *predictive attentional saccades* [19]. For example, one could add to the address of the current winner an offset based on the mean speed of the nut.

### 5.2. Overt Attention with Natural Moving Stimuli

The final experiment was performed with freely moving stimuli in front of the retina. At the beginning of the experiment the retina is still, and a covert FOA scan path is produced. Subsequently the motor movements are enabled, and the PTU is used to foveate the selected stimulus. In this behavioural condition we implement a form of saccadic suppression, preventing the DVS output to reach the SAC input during saccade execution; this mechanism avoids unnecessary and probably disruptive computation of FOA location during the fast movement of the sensor.

We tested the system with a person first waiving hands and then walking in the field of view of the DVS.

During the covert attention period (not shown), the SAC output oscillates between the locations of moving objects, in this case waiving hands. The SAC alternates between the two hands, while it selects only rarely the arms that are moving as well. This behavior confirms once more that stimuli with a dense and circumscribed region of activity, as the hands, have competitive advantage with respect to single edges, thanks to the lateral excitation.

**Figure 12.**
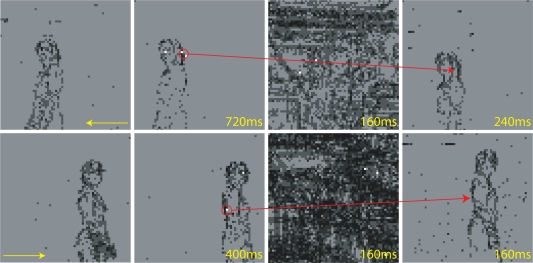
Overt attention with moving natural stimuli: two sequences of movement (yellow arrow), target selection (red circle), saccade movement and landing (red arrow), when a person is walking to the left (upper sequence) and to the right (lower sequence).

Figure 12 shows examples of sequences of target selection, saccade movement and landing, during the overt attention period (The chip’s activity is grouped into frames and displayed as a movie by the jAER - java AER - software tool designed by T. Delbrück. In the experiments shown in this section, the frame duration has been arbitrarily chosen to be 80 ms.). The SAC selects pixels belonging to the person’s contour and the system reliably follows the subject’s trajectory.

The speed of stimuli for which the system works is limited by the delays introduced by the software algorithm, the communication between the software and the hardware components, and the fixed duration of the saccadic suppression mechanism. After the saccade, the system waits until the end of saccadic suppression period to compute a new location for the next saccade. If the tracked stimulus moves too fast, it can disappear from the retina’s field of view, before the system is ready to select the new saccade target.

## 6. Conclusions

The two-chip selective attention system described in this paper is the first realization of a full-custom AER-based active-vision system, capable of sequentially selecting relevant regions of the input stimuli. The SAC performs non-trivial processing on the output activity of a neuromorphic silicon sensor (the DVS) and its output is used to drive a pan-tilt unit that orients the DVS toward the most salient region in the sensor’s field of view. The system design incorporates biologically inspired computing principles. The processing of sensory data is asynchronous and event-driven, implementing both efficient computation and communication. The specific computation performed by the SAC extracts relevant information via a cooperative/competitive mechanism that takes into account the relative context of the input, rather than the absolute value of each component (pixel). The implementation of adaptive mechanisms at the SAC input synapses, which models the short-term depression phenomenon observed in biological synapses, makes the system sensitive to transient stimuli and suppresses stationary stimuli.

The experiments described in this work demonstrate that the multi-chip system developed can reliably implement visual attention behaviors. The realization of a system with an actuator that closes the sensory-motor loop by orienting the sensor towards salient stimuli shows that the system can respond with an appropriate action to the sensory input stimuli. The last experiment described in this paper shows that the SAC output defines an unique winner, whose activity is stable for tens of milliseconds and can be directly used to control the actuators. The analysis of the system behavior when confronted with real world stimuli shows the effect of the dynamic interaction and the relative roles of its different components. Specifically we highlighted the role of short-term depression in the SAC input synapses, and showed that the SAC IOR mechanism is a crucial computational primitive required for proper system operation.

The actuation system used in this work comprises a motorized pan-tilt unit and a desktop computer running Matlab. At this “proof-of-principle” stage, the system’s set-up was not optimized for power consumption or speed. But the results obtained clearly demonstrate that such an approach can lead to an efficient and robust implementation of autonomous visual attention sensory-motor systems, for example by using micro-controllers or dedicated field programmable gate-array (FPGA) devices and boards, to replace the general purpose PCI-AER board and desktop.
